# Ellagic acid mediates cardioprotection against adrenaline-induced toxicity via PI3K/AKT and Keap1-NRF2 axes

**DOI:** 10.1038/s41598-025-21317-7

**Published:** 2025-10-20

**Authors:** Tahany Saleh Aldayel, Sahar A. Abou Haleka, Hala M. Ebaid, Noha M. Abd EL-Fadeal, Heba M. Abdelrazek, Heba N. Gad EL-Hak, Khaled M. Darwish, Hanan M. Rashwan

**Affiliations:** 1 Department of Health Sciences, Clinical Nutrition, College of Health and Rehabilitation Sciences, Princess Nourah bint Abdulrahman University, 11671 Riyadh, Saudi Arabia; 2 Department of Zoology, Faculty of Science, Suez Canal University, Ismailia, Egypt; 3 Department of Medical Biochemistry and Molecular Biology, Faculty of Medicine, Suez Canal University, Ismailia, Egypt; 4 Medical Biochemistry Unit, Ibn Sina National College for Medical Studies, Jeddah, Kingdom of Saudi Arabia; 5 Oncology Diagnostic Unit Faculty of Medicine, Suez Canal University, Ismailia, Egypt; 6 Department of Physiology, Faculty of Veterinary Medicine, Suez Canal University, Ismailia, 41522 Egypt; 7 Department of Medicinal Chemistry, Faculty of Pharmacy, Galala University, New Galala, 43713 Egypt; 8 Medicinal Chemistry Department, Faculty of Pharmacy, Suez Canal University, Ismailia, 41522 Egypt; 9 Department of Zoology, Faculty of Science, Arish University, North Sinai, Egypt

**Keywords:** Adrenaline, Ellagic acid, Oxidative stress, Inflammation, Rat model, Heart, Physiology, Biomarkers, Cardiology, Diseases

## Abstract

Ellagic acid (Ea) is an example of a bioactive polyphenolic compound with numerous beneficial effects; therefore, it is used to counteract adrenaline toxicity in this study. Thirty-six male rats were categorized into 6 groups of 6 rats. Group (1) was given oral purified distilled water for thirty successive days and injected with a saline for the next two days. Groups (2) and (3) received 7.5 and 15 mg/kg body weight Ea orally, followed by saline injection for two days. Group (4) was given distilled water orally for 30 uninterrupted days, followed by adrenaline injections for the next two days. Groups (5) and (6) received 7.5 and 15 mg/kg Ea for 30 days, followed by adrenaline injections for the next two days. Electrocardiogram (ECG) changes, oxidative stress, inflammation, immunohistochemistry, and histopathological alterations were evaluated. Specific biomarkers associated with kidney, liver, and heart injuries were recorded. At the higher dose, the Ea counteracted adrenaline-induced heart rate decrease, prolongation of the QT interval, and elevation of the ST interval in rats. It also enhanced kidney, liver, and heart function, ameliorating abnormal ECG patterns and tissue architecture changes. Ea suppressed PI3K/AKT signaling pathway and promoted nuclear factor erythroid 2-related factor 2 (NRF2) expression in the heart, possibly due to its antioxidative and anti-inflammation potential. Additionally, the study suggested a mechanistic aspect regarding the Ea’s antioxidant activity through modulating the Keap1-NRF2 axis based on a validated computational approach that warrants further investigation. This study highlights the potential benefits of Ea in reducing heart injury.

## Introduction

 The administration of exogenous adrenaline disrupts the body’s normal homeostasis^1^. This drug is highly effective and commonly used in emergencies due to its potent activity. It has been utilized to treat cardiac arrest resuscitation, anaphylactic reactions, and acute asthma exacerbations due to its potent cardiostimulatory and vasoconstrictive actions^2^. Nonetheless, administration of adrenaline at excessive doses or over prolonged periods has been associated with significant detrimental outcomes affecting various organ systems^3^. Documented complications include myocardial ischemia and arrhythmias attributable to increased oxygen consumption and electrical instability as well as hypertension-induced target-organ damage; hepatic dysfunction; and renal impairment that primarily result from heightened oxidative stress responses^4^. Experimental studies on cardiac arrest have demonstrated that administering a high dose of adrenaline, as opposed to the standard dosages of 0.01–0.02 mg/kg, enhances myocardial and cerebral blood flow, improves the equilibrium between myocardial demand and oxygen supply, and increases resuscitation rates^5^. Several uncontrolled clinical trials have assessed high doses of adrenaline as a consequence of these investigations. In the study of Stiell, Hebert^6^, it was noticed that administering high adrenaline doses significantly improved in coronary perfusion pressure, arterial diastolic pressure, resuscitation rates, and neurological outcome. Long-term studies have demonstrated the adverse influence of adrenaline on the cardiovascular system, especially the heart^7^. Cardiotoxic adrenaline induced effects have been noticed in animal models^8^. Efforts to enhance myocardium to overcome adrenaline induced toxicity have yielded limited success. Hence, there is a need to create a therapeutic plant supplement that can effectively mitigate the toxic effects caused by adrenaline. Given this spectrum of clinically meaningful toxicities arising from adrenergic overstimulation which may occur inadvertently through dosing errors or be part of disease processes. Comprehensive experimental endpoints pertinent both mechanistically and translationally, were selected. Non-invasive ECG provides sensitive insight into electrophysiologic disturbances paralleling those encountered during catecholamine surges in human patients^9^. Cardiac injury biomarkers including CKMB/LDH reliably indicate myocyte membrane compromise akin to changes observed following infarction^10^. Liver transaminases (ALT/AST) assess hepatotoxicity while serum urea/creatinine levels monitor nephrotoxicity consistent with systemic side-effects documented after inappropriate adrenaline use^11^. Indices reflecting antioxidant capacity, lipid peroxidation markers, along with inflammatory cytokines provide additional understanding regarding redox imbalance mechanisms driving tissue damage^12^. Finally, analyses focusing on molecular regulators such as elements within the PI3K-AKT-NRF2 pathway allow us not only to characterize toxicity but also elucidate possible protective strategies exerted by bioactive compounds under investigation^13^.

Ellagic acid (Ea) is an inherent compound occurring in varieties of food items, berries, and nuts. It can also be found in other sources, such as pomegranate extract, where it is highly concentrated^14^. Ea is a naturally occurring polyphenol with anti-inflammatory and antioxidant properties^15^. Antioxidant polyphenols possess potent oxidizing properties, making them advantageous in inhibiting cardiovascular diseases^16^. New studies have shown that Ea and their derivatives significantly impact the cardiovascular system^17^. Moreover, Ea can decrease the extent of tissue damage in a model of myocardial infarction and the duration of tissue damage following prompt intervention^18^. Ea treatment can reduce the rate at which the heart rate changes and lower the levels of interstitial creatine kinase, leading to improved in left ventricular developed pressure (LVDP) recovery. Ea exhibits an inhibitory effect on fibrotome activity in heart failure induced by doxorubicin, as well as displaying antihypertrophic effects^19^. Furthermore, Ea inhibits heart failure caused by tachycardia^20^. All the studies indicate that Ea has a cardioprotective effect and provides a distinct advantage in the realm of cardiovascular health. The precise impact of Ea on the injury caused by adrenaline is not widely understood, and the exact molecular machinery implying its cardioprotective potential persists indefinitely. While previous studies have explored various polyphenols as cardioprotective agents through antioxidant mechanisms, the existing literature presents notable gaps. Prior investigations of Lecour and Lamont^21^ have primarily focused on isolated aspects of polyphenol activity in different cardiac injury models. Ajzashokouhi, Rezaee^22^ demonstrated that certain polyphenols modulate the PI3K/AKT pathway in ischemia-reperfusion models, while Fakhri, Moradi^23^ showed NRF2 activation by flavonoids in pressure-overload heart failure. However, these studies examined different pathological contexts, utilized diverse polyphenolic compounds, and typically assessed either oxidative stress pathways or inflammatory cascades in isolation. Our investigation is distinct in several critical aspects as it specifically examines Ea’s protective effects in the clinically relevant context of adrenaline toxicity a condition with unique pathophysiological features; it simultaneously investigates both PI3K/AKT suppression and Keap1-NRF2 axis activation within the same experimental model; it employs a comprehensive multi-organ assessment rather than focusing solely on cardiac parameters; and it integrates computational validation to elucidate the molecular interaction between Ea and the Keap1-NRF2 complex. This multifaceted approach provides novel insights into how Ea’s molecular actions translate into functional cardioprotection against catecholamine surge, addressing a significant gap in therapeutic options for this specific form of cardiac injury.

The existing investigation sought to probe the cardioprotective influence of Ea against adrenaline- inflicted harm in a rat model and elucidate its underlying molecular mechanisms. The investigation specifically focused on evaluating the dose-dependent effects of Ea (7.5 and 15 mg/kg) on cardiac function through a comprehensive assessment of ECG parameters, cardiac biomarkers, and histopathological alterations, while simultaneously examining its multi-organ protective capabilities by analyzing kidney and liver function parameters along with tissue architecture modifications. Furthermore, the study sought to delineate the molecular mechanisms underlying Ea’s protective effects through investigation of the phosphoinositide 3-kinases (PI3K)/protein kinase B (AKT) signaling pathway, analysis of nuclear factor erythroid 2-related factor 2 (NRF2) expression and stimulation, assessment of oxidative stress markers, and evaluation of inflammatory mediators. It was complemented by computational validation of the mechanistic interplay between Ea and the Keap1-NRF2 axis. This integrated approach aimed to provide a thorough understanding of Ea’s therapeutic potential in mitigating adrenaline-induced cardiac injury and its broader implications for organ protection.

## Materials and methods

### Animals

Thirty-six adult male albino rats from the Wistar type (140–160 g and four months of age) were residing in clean polyethylene plastic cages. The animals were housed at room temperature, provided unrestricted access to tap water, and fed standard laboratory rat food. Before being utilized in the present experiment, the rats underwent a one-week acclimation period to familiarize themselves with the laboratory settings.

### Chemicals

Sigma-Aldrich Chemical Co., located in St. Louis, MO, USA, supplied Ea ≥ 95% (HPLC) with CAS Number: 476-66-4 and all substances and reagents used in this investigation. Ea was administered orally as a suspension in distilled water. Although EA exhibits limited solubility in water (~ 0.8 µg/mL at neutral pH), the compound was finely ground and sonicated to ensure uniform dispersion. This method aligns with prior preclinical studies demonstrating detectable plasma and tissue levels of EA after oral dosing in rodents^24^.

### Experimental design

The experimental rats were categorized into six groups (*n* = 6) according to Sadiq, Usman^25^: Group (1) (Ctrl) orally administered distilled water for thirty consecutive days, after which saline was i.p. injected for two days. Groups (2) (Ea-L) and (3) (Ea-H) were orally administered Ea at 7.5 and 15 mg/kg bwt doses, respectively, for 30 consecutive days according to Kannan and Quine^26^ and Najjar and Feresin^27^ in which similar dosing regimens demonstrated efficacy and safety in rodent models investigating cardioprotective or anti-inflammatory effects. These selected doses within a therapeutically relevant and non-toxic range. A saline injection for 2 days followed this. Group (4) (Adr) orally administered distilled water for 30 consecutive days, after which they received adrenaline injections (2 mg/kg) for 2 days (1 mg/kg each day) according to Haleka, Rashwan^28^. Groups (5) (Ea-L + Adr) and (6) (Ea-H + Adr) were orally administered Ea at 7.5 and 15 mg/kg bwt doses, respectively, for 30 consecutive days, after which they received adrenaline injections (2 mg/kg) for 2 days (1 mg/kg each day). Additionally, all experimental protocols adhere to the ARRIVE (Animal Research: Reporting of In Vivo Experiments) guidelines for reporting animal research. The animal study protocol was approved by the Faculty of Science, Suez Canal University, Ismailia, Egypt (approval number: REC348/2024) for studies involving animals. all methods were performed in accordance with the relevant guidelines and regulations. All experimental procedures adhere to the ARRIVE guidelines and were performed following the recommendations of the National Institutes of Health Guide for Care and Use of Laboratory Animals.

### Electrocardiography (ECG) examination

ECG charts were recorded on the 33rd day of the experiment (24 h after the last adrenalin injection). Tetrahydrofuran (> 99%, Sigma-Aldrich Cat#186562, USA) was administered to the animals to achieve full anesthesia prior to placing them on their back for ECG recording. Electrodes were applied under the rat’s skin and allied to the Kaden YasenTM ECG-903 device. In order to analyze changes in heart rate, QT interval, and ST-segment, a Lead II electrocardiogram (ECG) was done following the procedures designated by Youssef, Abdelrazek^29^.

### Samples collection and preparation

Upon completion of the experiment, Retroorbital blood samples were collected via sterile capillary tubes under tetrahydrofuran-induced inhalation anesthesia. The sample of blood was collected and promptly subjected to centrifugation at 4000 revolutions per minute for 15 min. The sera samples were isolated, gathered, and preserved at −80℃ for biomarkers analyses.

The hearts were dissected and partitioned into three segments. Part of the heart was kept at −80 °C to prepare heart homogenates. The second piece of the heart was preserved in a solution of buffered formalin (Thermo Fisher, USA) for using in histopathological and immunohistochemical analyses. The third piece of the cardiac tissue was quickly frozen at −80 °C for use in the extraction of total RNA and the analysis of real-time RT-PCR of PI3K and AKT expression. For cardiac tissue homogenates preparation, 10 mg of tissue were scrubbed with solution of phosphate buffer and then homogenized within 1 mL of the same solution. The resulting mixture was stored at −20 °C overnight. Following the completion of 2 freeze-thaw cycles to disrupt the plasma lemma of cells, centrifugation of the homogenates for 5 min at 5000 x g and 2–8 °C temperature range. The liquid portion was extracted and promptly examined for biochemical analyses.

### Body weight and changes in organ weight

The body weight of each rat was recorded by means of precise balance during the acclimatization period, once prior to the commencement of experiment (at start of dosing) and the last time on the sacrifice day. The kidneys, liver, and heart were isolated, and their weights were obtained following sacrifice. The ratio of organ weight to body weight was computed following the methodology designated by Zhang, Zhai^30^.

### Organ function biomarkers

#### Biomarker of heart injury

The concentration of lactate dehydrogenase (LDH) in sera was measured via the Spectrum commercial kit (Cat. NO. 278001) from Egypt, following the procedures illustrated by Gad El-Hak, Mahmoud^31^. The concentration of creatine kinase-MB (CK-MB) in the blood serum was measured using MyBioSource, USA (Cat. NO. MBS2515061) ELISA kit. The kit had a detection range of 31.3–2000 pg/mL, as described by Altamimi, Alfaris^32^.

#### Biomarkers of kidney injury

The Bjornsson^33^ method was employed to determine the serum creatinine. The concentration was determined using spectrophotometry at a wavelength of 492 nm. The measurement of serum creatinine was typically reported in milligrams per deciliter (mg/dL). The methodology illustrated by Shrestha, Gyawali^34^ was employed to determine the serum urea levels. The concentration was determined by using spectrophotometry at a wavelength of 578 nm. The measurement of serum urea was reported in milligrams per deciliter (mg/dL). The measurement of serum uric acid was conducted using the Zhao, Yang^35^ methodology. The concentration was quantified by using spectrophotometry at a wavelength of 550 nm. The measurement of serum uric acid was reported in milligrams per deciliter (mg/dL).

#### Biomarker of liver injury

The concentration of aspartate aminotransferase (AST) in blood serum was measured using Biovision, UK, (Cat. NO. E4321-100) ELISA kit with 0.31- 20 ng/mL detection range, as described by Ahn, Bae^36^. The measurement of alanine aminotransferase (ALT) was conducted via the methodology mentioned by Yang, Park^37^. The activity of alkaline phosphatase (ALP), measured in U/L, was determined implementing the procedures mentioned by Kaplan^38^. The concentration of total protein, estimated in grams per deciliter (g/dL), was measured via the method illustrated by Guo, Zebda^39^.

#### Cholesterol and triglycerides levels

The concentration of cholesterol, measured in mg/dL, was determined using the method developed by Artiss and Zak^40^ methods. The Czerny, Pawlik^41^ methodology was employed to estimate the triglycerides levels. The concentration of serum triglycerides that was reported in mg/dL was determined using spectrophotometry at a wavelength of 546 nm.

### Inflammation and oxidative stress

#### Biomarkers of heart inflammation

The concentration of interleukin-6 (IL-6) in the heart homogenate was measured using an Abcam, UK (Cat. NO ab242490) ELISA kit that had a detection range of 15.6 to 1000 pg/mL, as described by Savale, Tu^42^. The concentration of interleukin-1β (IL-1β) in the heart homogenate was measured using Cusabio Technology, USA (Cat. NO CSB-E08055r) ELISA kit with 0.156-10 ng/mL detection range^43^. The content of tumor necrosis factor-alpha (TNF-ɑ) in the heart homogenate was estimated using Cusabio Technology, USA (Cat. NO TA CSBE11987r) ELISA kit with a range of 6.25 to 400 pg/mL for detection.

#### Biomarkers of oxidative damage

The estimation of serum total antioxidant capacity (TAC) was conducted via the procedures mentioned by Hassler, Almer^44^. The level was calculated by extrapolating the total organic carbon (TOC) calibration curve at 450 nm. The concentration of reduced glutathione in blood was measured using MyBioSource (Cat. NO. MBS265966, USA) ELISA commercial kit with 100 − 1.56 ug/mL detection range, according to AlFaris, Alshammari^45^. The biomarker malondialdehyde (MDA), which indicates lipid peroxidation, was measured in the heart homogenate using Biodignostic, Egypt, (Cat. NO.MD 2529, USA) following the procedures mentioned by El-Naseery, Mousa^46^. The serum total oxidative stress (TOS) was quantified utilizing the method developed by Jansen and Ruskovska^47^. The concentration was determined by extrapolating the TOS calibration curve at a wavelength of 510 nm.

### Molecular assessments

#### Cardiac PI3K and AKT 1 expression by RT-PCR

The frozen left ventricular tissues were subjected to RNA extraction (PureLink^®^ RNA Mini kit, Invitrogen, USA) as mentioned in the manufacturer’s enclosed pamphlet. Quantification of the extracted RNA was proceeded using a Qubit^®^ Fluorometer, USA. The concentrations of RNA in the samples were adjusted to be 200 ng/L, and cDNA was synthesized via Thermo Fisher Scientific, Inc., USA Reverse Transcription kit. Gene expression was analyzed using RT-qPCR. To identify variations in gene expression, we used SYBR^®^ Select Master Mix (manufactured by Applied Biosystems, US) along with specific primers. The sequences of the used primers are demonstrated in Table 1. The expression data was normalized using the B-actin mRNA fragment as a housekeeping gene. The gene expressions were calculated by determining the relative fold change compared to the control group.

**Table 1:** The details of primer sequences for the genes amplified according to Palabiyik, Tastekin^48^.

Genes	Forward primer sequence (5’−3’)	Reverse primer sequence (5’−3’)
PI3K	CATGGATGCTTTGCAGGGTTT	CCAGATGTTCTCCATGATTC
AKT1	ACTGACATTGGACGGCTGAG	CAGGTGGGACTGTGATACGG
ß-actin	AGAGGGAAATCGTGCGTGAC	CAATAGTGATGACCTGGCCGT

### Histology

The formalin-preserved heart tissue was treated using the conventional histological techniques described by Fischer, Jacobson^49^. The HX&E-stained slides were analyzed and captured by a microscope equipped with a digital camera. The stained slides of Cardiac sections from each animal were examined and scored independently by two experienced pathologists who were blinded to group assignments. Necrosis and inflammation was graded on a scale of 0–3: *0* = absent; *1* = mild (< 25% tissue); 2 = moderate (25 to 50%); 3 = severe (> 50%) according to Smith, Lightfoot^50^.

### Immunohistochemistry for NRF2

The paraffin-embedded slices underwent xylene deparaffinization and were then hydrated using ethanol. The antigen retrieval process utilized. Staining was applied to the cardiac sections following the manufacturer’s instructions: “EnVision TM FLEX horseradish peroxidase labelled. A dilution 1:100 of the primary polyclonal NRF2 antibody Polyclonal anti-NRF2 antibody (ABCAM #ab206890; UK) was employed in a phosphate buffer saline (PBS) solution. The primary antibody corresponding to the target was left to incubate on the sections overnight. The dyeing was done by utilizing DAB substrate chromogen after conjugating dextran with peroxidase molecules and goat secondary antibody molecules that are immunoglobulins specific to rabbits. Subsequently, the area that had been previously stained was subjected to staining with Mayer’s hematoxylin. NRF2-positive cytoplasm staining was quantified as the percentage (%) of positively-stained cardiomyocytes within five randomly selected high-power fields per slide using ImageJ software.

### Statistical analysis

Data normality and homogeneity of variance were assessed prior to conducting parametric analyses using the Shapiro-Wilk and Levene’s tests, respectively. For normally distributed data, one-way ANOVA followed by Tukey’s post-hoc test was employed for multiple group comparisons. The values are reported as means ± standard error of the means (SE). A statistically significant difference was observed when the p-value fell below 0.05.

### Molecular modelling study

Atomic coordinates of Keap1 biotarget were retrieved from RCSB PDB from the following codes; PDB ID: 7P5N, for subsequent preparation and minimization under AMBER/partial charges modified forcefields using the Auto Dock software v1.2.0 as per the reported studies^51,52^. The binding site was delineated to endorse the co-crystallized ligand; pyrazole carboxylate-based Keap1 kelch domain’s NRF2 interaction inhibitor (CHEMBL5199314)^53^. The docking grid-box was set at dimensions (20 × 20 × 20 Å^3^) to enclose all the reported key binding residues at the Keap1 pockets. Molecular docking protocol was then proceeded through AMBER Forcefield and Lamarckian/Genetic Algorithm to generate the investigated ligands’ poses^54^. The docking parameters were established with an exhaustiveness of 100, 20 iterated binding modes, and a maximum free-binding energy difference of 4 Kcal/mol between the predicted binding modes^55^. The best predicted pose was selected in terms of high docking scores, low root mean-squared deviation; RMSD < 2.00Å cut-offs from ligand, and/or depicted contacts with reported key pockets residues being consent with small molecule kinase inhibitors. The application of PyMol v2.0 facilitated the visualization of molecular modeling results and enabled the analysis of compound-kinase interactions and conformations.

## Results

### The ratio of organ weights to body weight

The group treated with adrenaline exhibited a statistical increase (*p* = 0.000; *P* ≤ 0.05) in the ratio of kidney and liver weights to the body weight matched to the control group. Administration of Ea at doses of 7.5 and 15 mg/kg prior to adrenaline injection led to a statistically significant (*p* = 0.000; *P* < 0.05) decline in the ratios of kidney and liver weights to body weight matched to the group injected with adrenaline. Heart relative weights exhibited non-statistical variation among groups (Table 2).

**Table 2:** Influences of pretreatment with ellagic acid (Ea) on body weight and changes in relative organs’ weight in adrenaline injected male Wistar rats.

	Initial weight (g)	Final weight (g)	Kidney (%)	Liver (%)	Heart (%)
Group (1)	164.30 ± 1.61 ^a^	251.75 ± 1.29 ^a^	0.703 ± 0.02 ^b^	3.39 ± 0.04^d^	0.351 ± 0.01 ^a^
Group (2)	159.70 ± 1.29 ^a^	222.25 ± 5.11 ^ab^	0.709 ± 0.01^bc^	3.400 ± 0.002 ^d^	0.345 ± 0.001^a^
Group (3)	153.00 ± 7.21 ^a^	211.00 ± 5.30 ^b^	0.705 ± 0.04 ^b^	3.345 ± 0.03 ^d^	0.344 ± 0.002 ^a^
Group (4)	161.32 ± 3.91^a^	226.40 ± 4.71 ^ab^	0.820 ± 0.02^a^	4.21 ± 0.02^a^	0.360 ± 0.01^a^
Group (5)	165.00 ± 4.50 ^a^	226.83 ± 3.60 ^ab^	0.754 ± 0.01 ^c^	3.96 ± 0.02 ^b^	0.349 ± 0.002 ^a^
Group (6)	164.83 ± 3.52 ^a^	216.67 ± 7.0^ab^	0.721 ± 0.02 ^bc^	3.88 ± 0.015 ^c^	0.347 ± 0.001 ^a^
ANOVA	F(5,30) = 1.379, *p* = 0.234	F(5,30) = 5.099, *p* = 0.000	F(5,30) = 17.284, *p* = 0.000	F(5,30) = 391.766, *p* = 0.000	F(5,30) = 3.871, *p* = 0.002

Values are expressed as mean ± SEM (*n* = 6 per group). Superscript letters (a, b, c) indicate statistically significant differences between groups as determined by one-way ANOVA followed by Tukey’s post-hoc test (*p* < 0.05). Groups sharing the same letter are not significantly different. Group (1) (Ctrl). Groups (2) (Ea-L) and (3) (Ea-H). Group (4) (Adr). Groups (5) (Ea-L + Adr) and (6) (Ea-H + Adr).

### ECG changes

**Fig. 1:**
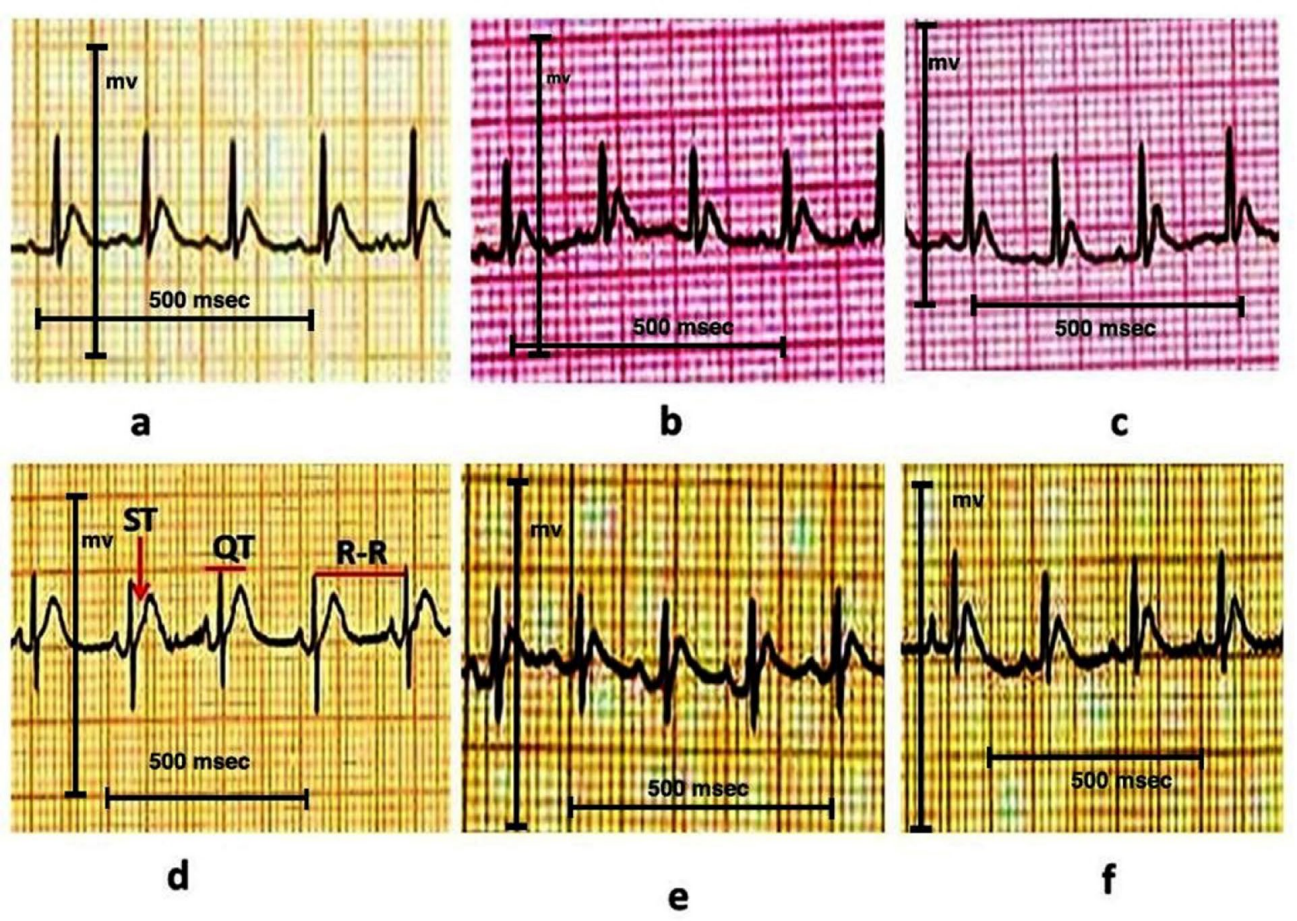
The effects of pretreatment with ellagic Acid (Ea) on the ECG pattern in adrenaline-injected male Wistar rats. (a): Group 1 (Ctrl) received oral distilled water for 30 consecutive days, followed by saline injections for the next two days. (b&c): Groups 2 (Ea-L) and 3 (Ea-H) were administered 7.5 mg/kg and 15 mg/kg body weight of Ea orally, respectively, followed by saline injections for two days. (d): Group 4 (Adr) received oral distilled water for 30 consecutive days, followed by adrenaline injections for the next two days. (e&f): Groups 5 (Ea-L + Adr) and 6 (Ea-H + Adr) were treated with 7.5 mg/kg and 15 mg/kg of Ea for 30 days, followed by adrenaline injections for the next two days. (ST segment: Represents the period between ventricular depolarization and repolarization. It starts from the end of the QRS complex and ends at the beginning of the T wave. QT interval: Measures the total time for ventricular depolarization and repolarization. It extends from the beginning of the Q wave to the end of the T wave. RR interval: Represents the time between two consecutive R waves (the peaks of the QRS complexes).

Heart rates, QT intervals, and ST elevation were non significantly altered in Ea (at dosages of 7.5 mg and 15 mg/kg) and Group (1). In comparison to the Group (1), the adrenaline injection produced a statistical (*P* = 0.000; *P* ≤ 0.05) decline in heart rate while increasing the QT interval and ST segment displacement. On the other hand, Ea and adrenaline groups co-administered groups displayed significantly higher heart rates, decreased QT 1intervals, and decreased ST segment displacement than rats treated with adrenaline (Table 3; Fig. 1).

**Table 3:** Influences of pretreatment with ellagic acid (Ea) on ECG pattern in adrenaline injected male Wistar rats.

	Heart rate (bpm)	QT interval (ms)	ST segment (mv)
Group (1)	360.2 ± 4.2^a^	34.11 ± 0.39 ^c^	0.038 ± 0.002 ^c^
Group (2)	361.2 ± 4.4^a^	33.71 ± 0.52 ^c^	0.035 ± 0.002 ^c^
Group (3)	358.7 ± 8.6^a^	33.02 ± 0.72^c^	0.032 ± 0.002 ^c^
Group (4)	225.2 ± 4.6^b^	56.13 ± 1.45 ^a^	0.110 ± 0.003 ^a^
Group (5)	327.6 ± 16.6^a^	44.91 ± 2.70 ^b^	0.081 ± 0.005 ^b^
Group (6)	331.8 ± 15.7^a^	41.21 ± 1.12 ^b^	0.070 ± 0.003 ^b^
ANOVA	F(5,30) = 14.38, *p* = 0.000	F(5,30) = 25.860, *p* = 0.000	F(5,30) = 47.958, *p* = 0.000

Values are expressed as mean ± SEM (*n* = 6 per group). Superscript letters (a, b, c) indicate statistically significant differences between groups as determined by one-way ANOVA followed by Tukey’s post-hoc test (*p* < 0.05). Groups sharing the same letter are not significantly different. Group (1) (Ctrl). Groups (2) (Ea-L) and (3) (Ea-H). Group (4) (Adr). Groups (5) (Ea-L + Adr) and (6) (Ea-H + Adr).

### Biomarker of oxidative damage and antioxidant biomarker

The changes in biomarkers of oxidative damage were shown for the groups in Table 4. Compared to the Group (1), the adrenaline-induced myocardial injury caused a significant upsurge in MDA and TOS. In contrast, a significant decline in GSH and TAC was noted compared to the rats that received adrenaline injections. Ea and adrenaline co-administered groups caused a considerable (*P* = 0.000; *P* ≤ 0.05) drop in MDA and TOS while promoted (*P* = 0.000; *P* ≤ 0.05) GSH and TAC.

**Table 4:** Influences of pretreatment with ellagic acid (Ea) on oxidative damage and antioxidant biomarkers in adrenaline injected male Wistar rats.

	MDA (nmol/mL)	TOS (mM/L)	GSH (µmol/L)	TAC (mM/L)
Group (1)	22.70 ± 1.12^a^	17.38 ± 1.31^a^	3.16 ± 0.05^a^	0.37 ± 0.02 ^a^
Group (2)	20.75 ± 0.9^a^	17.00 ± 1.71^a^	3.20 ± 0.12^a^	0.37 ± 0.01 ^a^
Group (3)	18.75 ± 1.3^a^	16.25 ± 1.3^a^	3.10 ± 0.09^a^	0.38 ± 0.01 ^a^
Group (4)	109.30 ± 5.2^b^	59.69 ± 3.25^b^	1.39 ± 0.17^b^	0.17 ± 0.03 ^b^
Group (5)	80.80 ± 4.7 ^c^	36.80 ± 1.42^c^	4.18 ± 0.24^c^	0.30 ± 0.005 ^c^
Group (6)	65.40 ± 5.9 ^c^	25.20 ± 1.53^c^	3.96 ± 0.15^c^	0.32 ± 0.009 ^a c^
ANOVA	F(5,30) = 64.191, *p* = 0.000	F(5,30) = 50.036, *p* = 0.000	F(5,30) = 33.338, *p* = 0.000	F(5,30) = 36.018, *p* = 0.000

Values are expressed as mean ± SEM (*n* = 6 per group). Superscript letters (a, b, c) indicate statistically significant differences between groups as determined by one-way ANOVA followed by Tukey’s post-hoc test (*p* < 0.05). Groups sharing the same letter are not significantly different. Group (1) (Ctrl). Groups (2) (Ea-L) and (3) (Ea-H). Group (4) (Adr). Groups (5) (Ea-L + Adr) and^6^ (Ea-H + Adr).

### Kidney injury biomarker

Table 5 showed that the mean creatinine, acid urea and uric levels across the groups. The injection of adrenaline resulted in significant promotion of these three parameters compared to the groups that were not injected with adrenaline. In comparison to the group that received adrenaline, the Ea and adrenaline co-administered groups showed a non-significant decline in the levels of creatinine, uric acid and urea except urea level in Group (6) (Ea 15 mg/kg + adrenaline) that was statistically lower than adrenaline group.

**Table 5:** Influences of pretreatment with ellagic acid (Ea) on kidney injury biomarkers in adrenaline injected male Wistar rats.

	Urea (mg/dL)	Creatinine (mg/dL)	Uric acid (mg/dL)
Group (1)	46.01 ± 0.86^a^	0.81 ± 0.01^a^	2.71 ± 0.05^a^
Group (2)	45.50 ± 1.19 ^a^	0.78 ± 0.03^a^	2.68 ± 0.10^a^
Group (3)	44.50 ± 1.04 ^a^	0.75 ± 0.03^a^	2.63 ± 0.18^a^
Group (4)	90.20 ± 1.01^b^	1.20 ± 0.01^b^	5.96 ± 0.11^b^
Group (5)	73.40 ± 1.63^b^	0.98 ± 0.02^b^	4.86 ± 0.37^b^
Group (6)	59.40 ± 3.20^c^	0.96 ± 0.02^b^	4.64 ± 0.22^b^
ANOVA	F(5,30) = 40.482, *p* = 0.000	F(5,30) = 13.356, *p* = 0.000	F(5,30) = 24.418, *p* = 0.000

Values are expressed as mean ± SEM (*n* = 6 per group). Superscript letters (a, b, c) indicate statistically significant differences between groups as determined by one-way ANOVA followed by Tukey’s post-hoc test (*p* < 0.05). Groups sharing the same letter are not significantly different. Group (1) (Ctrl). Groups (2) (Ea-L) and (3) (Ea-H). Group (4) (Adr). Groups (5) (Ea-L + Adr) and (6) (Ea-H + Adr).

### Liver injury biomarker, protein profile and lipid profile

Table 6 presented data of liver injury biomarker (AST, ALT, and ALP activities), protein profile (total protein and albumin), and lipid profile (levels of triglycerides and cholesterol) among the different groups. Ea-treated groups had non-significant AST, ALT, and ALP levels compared to Group 1. Adrenaline administration caused statistically significant (*P* = 0.000; *P* < 0.05) upsurge in AST, ALP, and ALT matched to Group (1). The pretreatment adrenaline group with Ea demonstrated a non-significant decrease in ALT and ALP. In contrast, a significant decrease (*P* = 0.000; *P* ≤ 0.05) in AST compared to the adrenaline group was observed.

The groups of control and rats that received Ea (7.5 mg or 15 mg/kg) revealed non-significant variation in albumin levels and total protein. Moreover, adrenaline injection caused a non-statistical (*P* = 0.140 and *P* = 0.009; *P* > 0.05) drop in albumin levels, while the total protein significantly declined. Pretreatment of adrenaline groups with Ea (7.5 or 15 mg/kg) resulted in non-significant increases (*P* = 0.43, *P* = 1.00; *P* > 0.05) in total protein and albumin as compared to rats that received adrenaline injections.

As indicated in Table 6, the levels of triglycerides and cholesterol in all experimental groups showed non-significant (*P* = 0.072 and *P* = 0.06; *P* > 0.05) variation.

**Table 6:** Influences of pretreatment with ellagic acid (Ea) on liver injury biomarker (AST, ALT, and ALP activities), protein profile (total protein and albumin) and lipid profile (levels of triglycerides and cholesterol) in adrenaline injected male Wistar rats.

	AST (U/L)	ALT (U/L)	ALP (U/L)	Albumin (g/dL)	Total protein (g/dL)	Cholesterol (mg/dL)	Triglycerides (mg/dL)
Group (1)	51.60 ± 1.3^a^	49.89 ± 2.12^a^	80.33 ± 2.14 ^a^	3.4 ± 0.1 ^a^	6.76 ± 0.2 ^ab^	59.27 ± 1.2 ^a^	45.01 ± 1.01^a^
Group (2)	50.50 ± 0.6^a^	49.25 ± 2.3^a^	82.50 ± 2.3^a^	3.50 ± 0.07 ^a^	5.98 ± 0.2 ^ab^	57.25 ± 2.5 ^a^	42.75 ± 1.7^a^
Group (3)	52.50 ± 1.0^a^	50.25 ± 3.5^a^	81.75 ± 4.1^a^	3.73 ± 0.09 ^a^	6.35 ± 0.2 ^a^	58.00 ± 3.1 ^a^	43.00 ± 1.4^a^
Group (4)	149.05 ± 5.7^b^	85.80 ± 3.5^b^	151.21 ± 3.1^b^	2.51 ± 0.30 ^a^	4.99 ± 0.5 ^b^	73.82 ± 1.19 ^a^	54.70 ± 1.11^a^
Group (5)	105.60 ± 3.5^c^	77.40 ± 2.1^b^	134.20 ± 1.9^b^	3.08 ± 0.15 ^a^	5.76 ± 0.2 ^ab^	68.20 ± 0.9 ^a^	51.40 ± 3.2^a^
Group (6)	102.60 ± 6.6^c^	74.00 ± 3.6^b^	127.80 ± 4.4^b^	3.18 ± 0.1 ^a^	5.84 ± 0.2 ^ab^	64.20 ± 3.2 ^a^	47.00 ± 1.3^a^
ANOVA	F(5,30) = 47.38, *p* = 0.000	F(5,30) = 19.354,*p* = 0.000	F(5,30) = 40.279, *p* = 0.000	F(5,30) = 1.643, *p* = 0.140	F(5,30) = 2.977, *p* = 0.009	F(5,30) = 2.373, *p* = 0.072	F(5,30) = 3.329, *p* = 0.06

Values are expressed as mean ± SEM (*n* = 6 per group). Superscript letters (a, b, c) indicate statistically significant differences between groups as determined by one-way ANOVA followed by Tukey’s post-hoc test (*p* < 0.05). Groups sharing the same letter are not significantly different. Group (1) (Ctrl). Groups (2) (Ea-L) and (3) (Ea-H). Group (4) (Adr). Groups (5) (Ea-L + Adr) and (6) (Ea-H + Adr).

###  Cardiac injury biomarkers and cardiac inflammatory cytokines

According to Table 7, serum LDH and CKMB activities significantly increased (*P* = 0.000; *P* ≤ 0.05) after adrenaline-induced myocardial damage in comparison to control rats. The oral pretreated adrenaline group with Ea resulted in a substantial drop (*P* = 0.000; *P* ≤ 0.05) in LDH and CKMB activities as matched to the adrenaline group.

Compared to the rats in the group (1), the data in Table 7 showed that the injection of adrenaline increased the levels of IL-1β, IL-6, and TNF-α. The observed increases were statistically varied (*P* = 0.000; *P* < 0.05).

Compared to rats that injected with adrenaline, the pretreatment of the adrenaline group with Ea significantly (*P* = 0.000; *P* ≤ 0.05) decreased cardiac IL-1β, IL6, and TNF-α levels.

**Table 7:** Influences of pretreatment with ellagic acid (Ea) on on cardiac injury biomarkers and cardiac inflammatory cytokines of adrenaline injected male Wistar rats.

	LDH (U/L)	CKMB (U/L)	IL-1β (ng/mL)	IL-6 (ng/mL)	TNF-α (ng/mL)
Group (1)	238.09 ± 10.12a	252.89 ± 15.11 a	0.435 ± 0.01a	0.759 ± 0.01a	0.797 ± 0.02a
Group (2)	222.75 ± 4.7 a	238.75 ± 12.7 a	0.420 ± 0.03a	0.750 ± 0.02a	0.800 ± 0.04a
Group (3)	241.00 ± 9.3a	245.50 ± 3.5 a	0.408 ± 0.02a	0.745 ± 0.03a	0.775 ± 0.02a
Group (4)	1489.30 ± 42.21b	1511.30 ± 52.11b	1.29 ± 0.04b	3.987 ± 0.04 b	3.91 ± 0.20 b
Group (5)	647.20 ± 9.7 c	734.60 ± 20.4 c	0.904 ± 0.03 c	3.580 ± 0.12 b	2.980 ± 0.05 c
Group (6)	495.20 ± 31.1 c	624.00 ± 18.5 c	0.572 ± 0.01 d	2.940 ± 0.12 c	2.260 ± 0.14 d
ANOVA	F(5,30) = 276.2, *p* = 0.000	F(5,30) = 276.2, *p* = 0.000	F(5,30) = 72.300, *p* = 0.000	F(5,30) = 158.33, *p* = 0.000	F(5,30) = 183.131, *p* = 0.000

Values are expressed as mean ± SEM (*n* = 6 per group). Superscript letters (a, b, c) indicate statistically significant differences between groups as determined by one-way ANOVA followed by Tukey’s post-hoc test (*p* < 0.05). Groups sharing the same letter are not significantly different. Group (1) (Ctrl). Groups (2) (Ea-L) and (3) (Ea-H). Group (4) (Adr). Groups (5) (Ea-L + Adr) and (6) (Ea-H + Adr).

### Cardiac PI3K and AKT expression

**Fig. 2:**
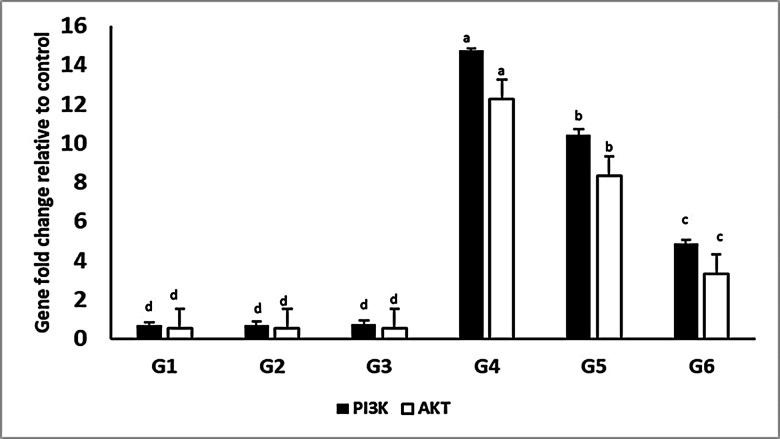
Effects of pretreatment with ellagic acid (Ea) on protein expression of PI3K and AKT. The data is displayed as the mean value plus or minus the standard error of the mean (SEM). Group (1) (Ctrl). Groups (2) (Ea-L) and (3) (Ea-H). Group (4) (Adr). Groups (5) (Ea-L + Adr) and (6) (Ea-H + Adr).One-way ANOVA is employed for statistical analysis, subsequently followed by Tukey’s multiple comparison test. The letters assigned to various means within the same column denote significant differences, as established by a one-way ANOVA test and subsequent Post Hoc Tukey’s test.

Compared to the rats in the control group, the injection of adrenaline caused a considerable rise (*P* ≤ 0.05) in the cardiac PI3K and AKT mRNA. The administration of Ea with adrenaline resulted in a statistical (*P* ≤ 0.05) reduction of PI3K and AKT protein expressions matched to the adrenaline injected group. The pretreatment with Ea 15 mg/kg produced statistically (*P* ≤ 0.05) lower PI3K and AKT protein expressions than 7.5 mg/kg dose (Fig. 2).

### Histopathological study

**Fig. 3:**
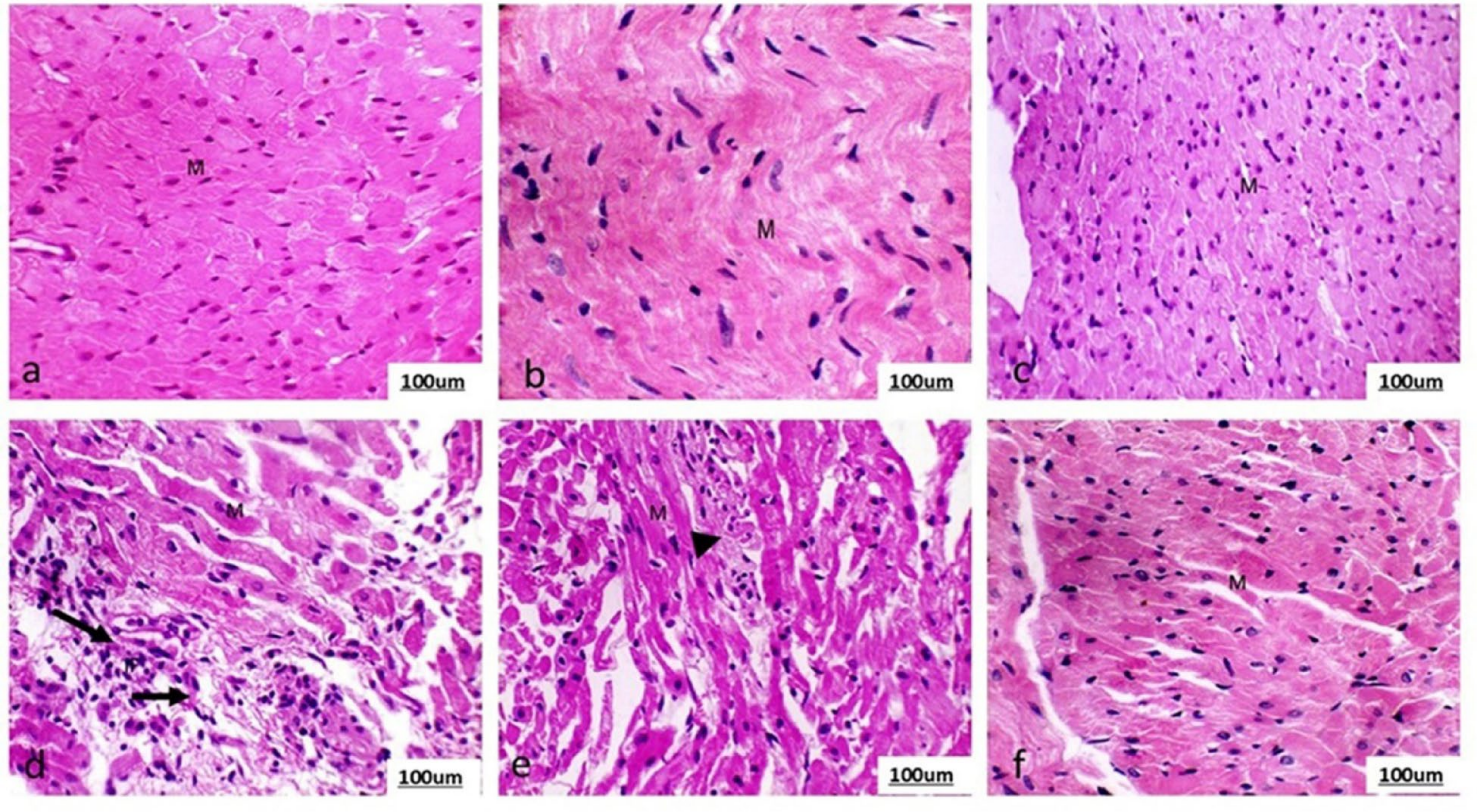
Photomicrograph of the heart section of (a) Group (1) (Ctrl) (b) Groups (2) (Ea-L) (c) Groups (3) (Ea-H) showed normal cardiac muscle fibers (M) devoid of any indications of inflammation or necrosis. (d) Group (4) (Adr) showed deteriorated cardiac muscle fibers with histological alterations, including inflammatory reaction, edema, cardiac muscle fiber degeneration and necrosis (arrow). Groups (5) (Ea-L + Adr) showed the heart displayed edema, a focused necrotic region that had been infiltrated by inflammatory cells (head arrow) and degeneration of the cardiac muscle fiber. (f) Groups (6) (Ea-H + Adr) showed the normal appearance of the heart sections. (H&E, 400X, scale bar = 100 μm).

The group (1) and Ea (at low and high doses) groups showed normal cardiac muscle fibers without any necrosis or inflammatory response, as demonstrated in Fig. 3 (a, b, and c) and Table 8. On the other hand, the adrenaline group showed distinct and specific histopathological changes, indicating acute myocardial infarction. These changes included multiple areas of focal necrosis in the heart muscle cells, which appeared intensely eosinophilic. The cardiomyocytes that underwent necrosis were heavily invaded by inflammatory cells, as shown in Fig. 3 d; Table 8. The histopathological lesions showed a regression in the groups treated with adrenaline and Ea at a low dose. In these groups, the heart exhibited a focal necrotic area infiltrated by inflammatory cells Fig. 3e; Table 8. Conversely, the most notable enhancements were observed in the groups receiving high Ea and adrenaline doses. The heart appeared normal in most sections examined in these groups, as depicted in Fig. 3f; Table 8.

**Table 8:** Blinded semiquantative histopathology analysis of the cardiac tissue.

	Necrosis (0–3)	Inflammation (0–3)
Group (1)	0.17 ± 0.08e	0.21 ± 0.07d
Group (2)	0.25 ± 0.10d	0.28 ± 0.09d
Group (3)	0.20 ± 0.05d	0.23 ± 0.06d
Group (4)	2.83 ± 0.16a	2.95 ± 0.14a
Group (5)	1.12 ± 0.13b	1.34 ± 0.21b
Group (6)	0.45 ± 0.11c	0.62 ± 0.09c

The data is displayed as the mean value plus or minus the standard error of the mean (SEM). Group (1) (Ctrl). Groups (2) (Ea-L) and (3) (Ea-H). Group (4) (Adr). Groups (5) (Ea-L + Adr) and (6) (Ea-H + Adr). One-way ANOVA is employed for statistical analysis, subsequently followed by Tukey’s multiple comparison test. The letters assigned to various means within the same column denote significant differences, as established by a one-way ANOVA test and subsequent Post Hoc Tukey’s test.

### Assessment of NRF2 using immunohistochemistry

**Fig. 4:**
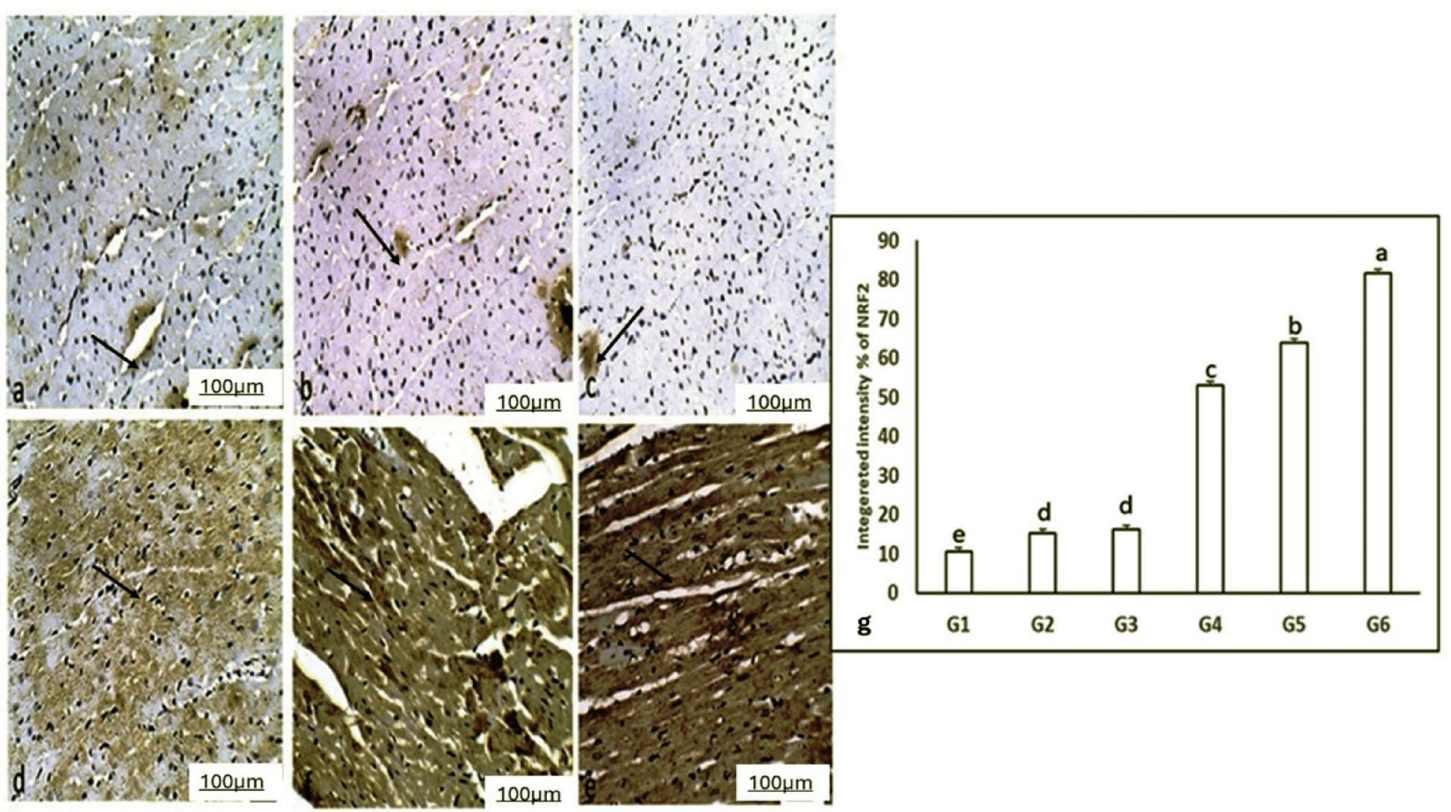
Photomicrograph of Heart immunohistochemical evaluations of the NRF2 pathway in both control and treated rats. (a) G1 (Ctrl) (b) G2 (Ea-L) (c) (Ea-H) (d) G4 (Adr) exhibited a moderate immune response of NRF2, as evidenced by the decrease of the present of the brown color in the cardiomyocytes (arrow). (e& f) G5 (Ea-L + Adr) and G6 (Ea-H + Adr) showed increase in the immunohistochemical expression of NRF2 immunostaining in cardiomyocytes (arrows). (H&E, 400X, scale bar = 100 μm). (g) histogram showed the percentage of integrated NRF2 intensity in the cardiac muscle. Data are presented as mean ± SEM. One-way ANOVA is employed for statistical analysis, subsequently followed by Tukey’s multiple comparison test. The letters assigned to various means within the same column denote significant differences, as established by a one-way ANOVA test and subsequent Post Hoc Tukey’s test.

Figure 4 displayed heart immunohistochemical evaluations of the NRF2 in both control and treated rats. The assessments revealed cytoplasmic brown coloration in the cardiomyocytes of the heart. The immunostaining of NRF2 in the hearts of normal and Ea rats exhibited severe level of immunohistochemical expression. The group treated with adrenaline exhibited a decreased immune response of NRF2, as evidenced by the reduction of the brown color in the cardiomyocytes. The concurrent administration of adrenaline and Ea, in both extract and dosage forms, elevated the immunohistochemical expression of NRF2 immunostaining in cardiomyocytes.

### Molecular modelling of Ea to potentially interfere with NRF2 modulation

**Fig. 5:**
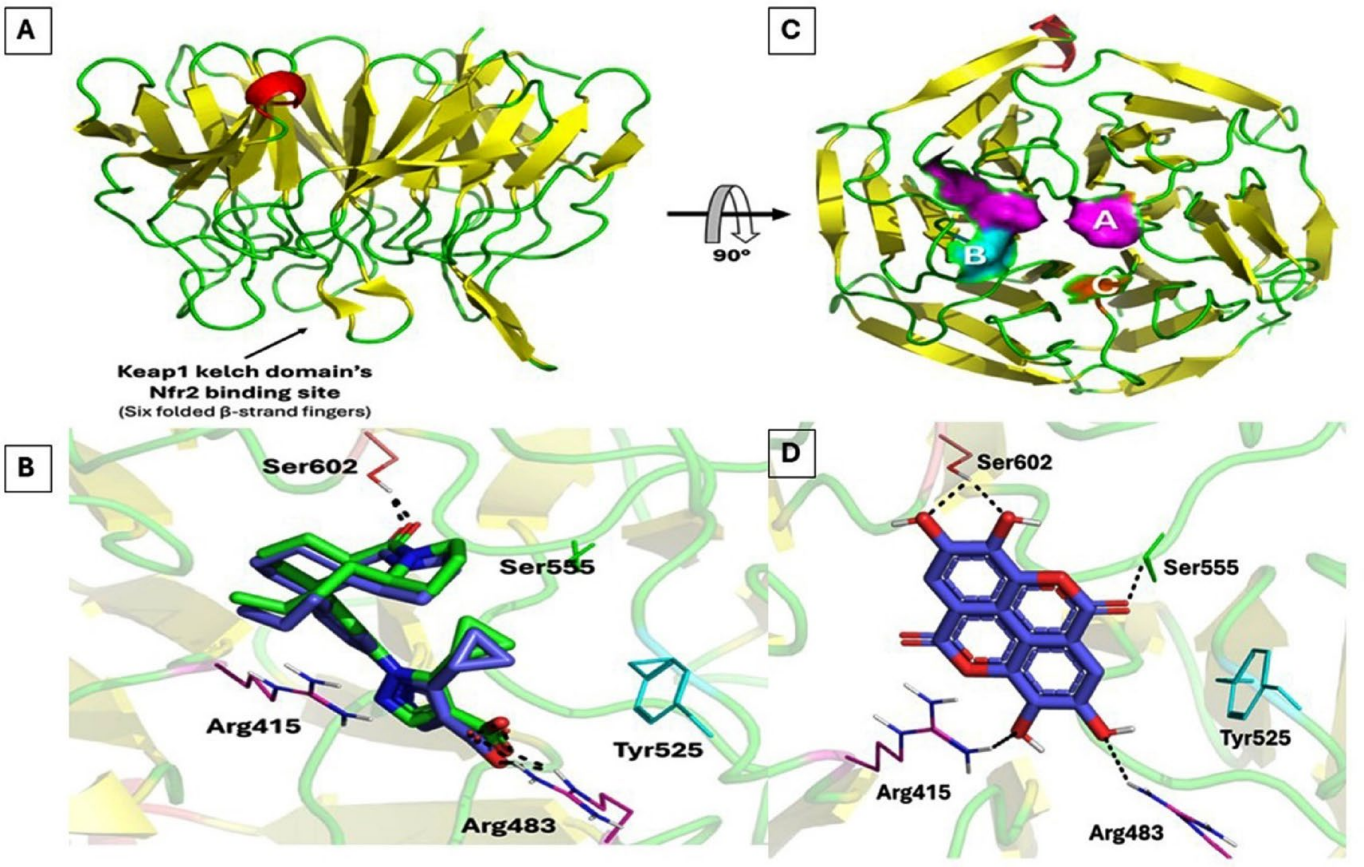
Keap1 kelch domain’s NRF2 binding site (PDB ID: 7P5N) and molecular docking results. (**A**) Structural features of Keap1 kelch domain shown in cartoon representation with the main secondary protein structures being colored differently. Side view shows the six folded β-strands of Keap1 kelch domain corresponding to NRF2’s ETGE or DLG conserved motif binding. Bottom view highlights the three hotspot sites; (A) electrostatic (Arg415 and Arg483; magenta), (B) Planar receptor (Tyr525; cyan), (C) hydrogen bond warhead (Ser602; orange). (**B**) Overlaid redocked non-covalent Keap1-NRF2 interaction small molecule inhibitor (blue) over its co-crystallized state (green); (**C**) Binding mode of Ea at Keap1 kelch domain’s NRF2 binding site. Ligands are presented as sticks and key binding residues as lines colored as corresponding protein secondary structure. Only key residues at 5 Å radius from ligand are shown and polar contacts are demonstrated as black-dash lines.

To further investigate the mechanistic aspects of Ea-NRF2 antioxidant activity, molecular modelling investigation was proceeded in computational predictions to explore the Ea’s potential to interfere with Kelch-like ECH-associated protein 1 (Keap1)/NRF2 interaction. Molecular docking simulation for Ea was performed at Keap1 crystallized domain’s NRF2 binding site (PDB ID: 7P5N) endorsing the co-crystallized Keap1/NRF2 interaction inhibitor, CHEMBL5199314^56^. Typically, the Keap1 kelch domain is a six-bladed β-propeller architecture being responsible for the recognition and binding to the NRF2’s structurally conserved DLG or ETGE motif^57,58^. This Keap1 domain folds into a shallow solvent-exposed and un-occluded by any relevant crystalline contacts which represents the canonical pocket for several reported non-covalent Keap1-NRF2 small molecule inhibitors^56^. Three main hotspot sites have been heavily reported at the Keap1 kelch domain, one with preference to the ligand’s electrostatic scaffold, another mediating hydrophobic contact with an aromatic core harboring a hydrogen bond acceptor (planar acceptor), and a final one resembling a polar warhead center with hydrogen bonding capability (Fig. 5A). Mimicking the NRF2’s Glu79 or Asp29 at ETGE or DLG motif, a ligand’s electrostatic scaffold has been recognized important for fulfilling the polar engagement with Keap1 Arg483 or Arg415, respectively^56,59,60^.

To initially validate the adopted docking protocol and algorism, redocking the co-crystallized pyrazole carboxylic acid-based inhibitor at the Keap1 Kelch domain depicted high conformational/orientation superimposition with a root-mean square deviation (RMSD) at 0.852Å (Fig. 5B). Typically, docking cogency for expecting both the energies and the binding modes at pertinent biological significance can be generally conferred with the ability of docking protocol to replicate the binding mode of co-crystallized inhibitor at RMSD < 2.00 Å^61–66^. Further, the redocked co-crystallized pyrazole inhibitor managed to replicate the compound-target interaction patterns including the ligand’s carboxylate‒Arg483 electrostatic salt bridge (1.7 Å/136.9°, 2.3Å/127.3°, and 1.7Å/166.3°), central core vans der Waal contact with Tyr525 (5.7Å), and terminal hydrogen bonding with Ser602 (1.7Å/153.9°) (Fig. 5B). Heightman (2019) et al. highlighted the importance of Bis-aryl scaffold within their lead KI696 inhibitor for mediating hydrophobic contacts with Keap1 Tyr525. Regarding our Ea’s docking simulation, the phytochemical molecule showed relevant anchoring at the Keap1 kelch domain significantly satisfying the binding requirements of the three key hotspot sites (Fig. 5C). Ellagic acid is an acidic phenol with a reported ability to exist within its anionic state (p*K*a.i. = 5.42 ± 0.01 and p*K*aii = 6.76 ± 0.01) under physiological conditions^67^. This was suggested to be beneficial for fulfilling the electrostatic demand of Keap1 Arg415 and Arg483 through the Ea’s predicted close-range docking polar interaction (2.9Å/124.1° and 3.0Å/150.2°, respectively). Notably, the central tetracyclic fused scaffold of Ea can be considered as a bis-aryl moiety with double cyclic ester groups connecting both sides of the aromatic rings. This Ea’s core cyclic ring scaffold was predicted useful to satisfy the non-polar contacts with Tyr525 at proximity (5.1Å). Finally, the terminal hydroxyl functionalities were suggested relevant as they mediated the strong double interactions with the last Keap1 hotspot, Ser602 sidechain residue (2.8Å/120.2° and 3.2Å/159.1°). Docking binding energy of Ea came just second to that of the redocked co-crystallized ligand at high-negative scores (7.48 Kcal/mol versus ‒8.26 Kcal/mol) highlighting significant Ea-Keap1 affinity and great potentiality for strong inhibitory profile.

## Discussion

**Fig. 6:**
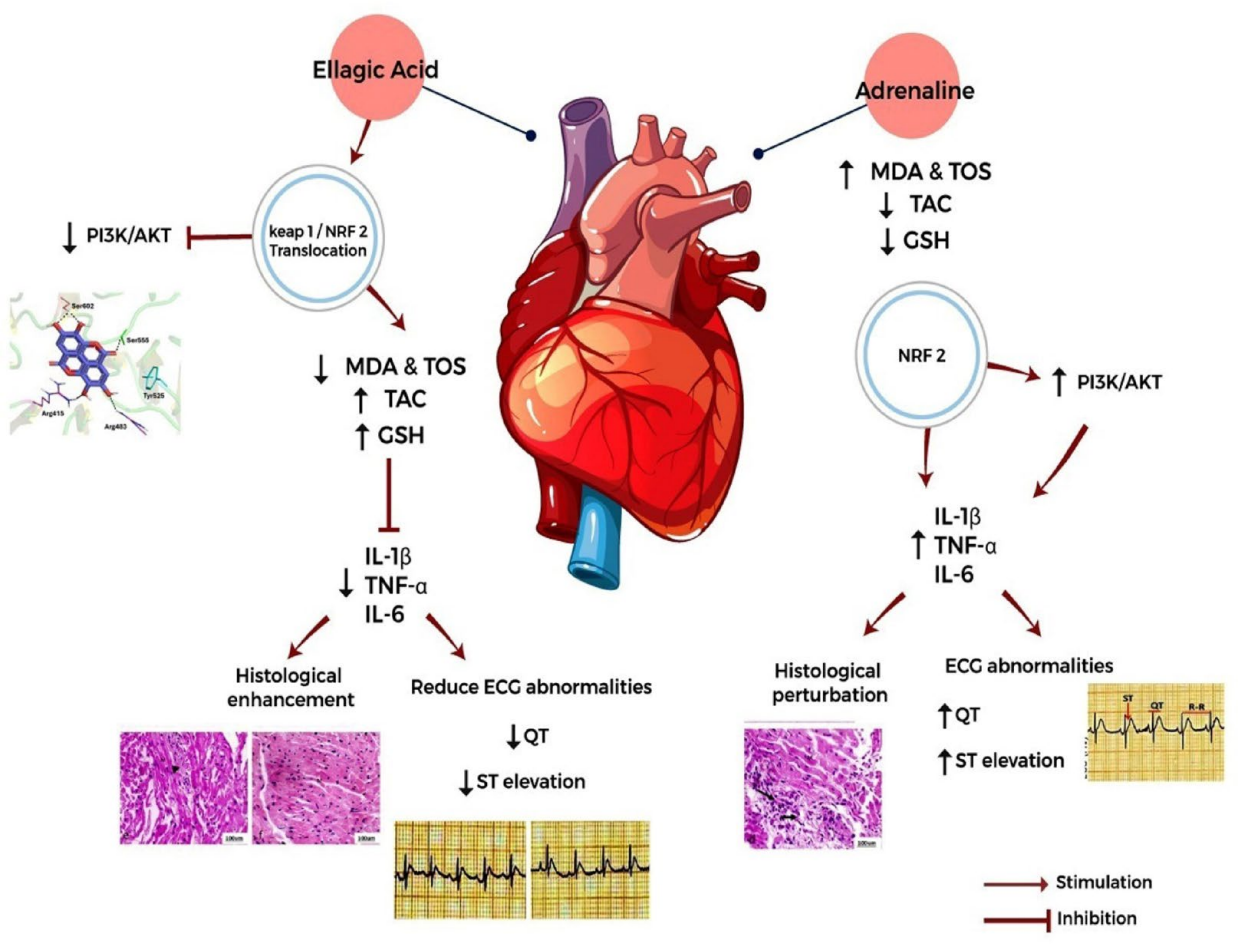
Summary figure of the effect of Ellagic acid and adrenaline.

The introduction of exogenous adrenaline injection is a highly successful approach for emergencies, including the treatment of cardiac arrest, despite its potential to alter the body’s homeostasis^68^. Ea is a bioactive polyphenolic compound^69^. It has long been recognized for its antioxidant and anti-inflammatory characteristics^70^. The present investigation examined the possible protective benefits of Ea against adrenaline-induced toxicity in rats, with a specific focus on the mechanism of cardio protection. This study demonstrates that adrenaline injections cause significant stress-induced alterations in multiple organ systems, notably the heart, kidneys, and liver, through mechanisms involving oxidative stress, inflammation, and organ dysfunction (Fig. 6). Pretreatment with Ea effectively ameliorated these effects, suggesting strong antioxidative, anti-inflammatory, cardioprotective, nephroprotective, and hepatoprotective properties.

The increased relative weights of the kidney and liver following adrenaline exposure, which may reflect inflammatory swelling or oxidative stress-induced damage, were in line with prior findings by Meyer, Stumvoll^71^ and supported by evidence of impaired hepatic protein degradation pathways^72^. Ea pretreatment normalized organ weights, likely due to its antioxidant capacity, as previously demonstrated by Kilic, Yeşiloğlu^14^. Interestingly, there was no significant change in heart weight, likely due to the brief duration of adrenaline administration, which is consistent with earlier findings noting insufficient time to affect cardiac mass^72^.

Cardiac function was assessed using ECG parameters, a key diagnostic tool for myocardial damage^73^. Adrenaline significantly affected ECG output, manifesting as decreased heart rate, QT prolongation, and ST segments displacement. These findings consistent with El-Marasy, El Awdan^74^ and Skulstad, Urheim^75^. These disruptions likely reflect ischemic changes and potential loss of cell membrane. Ea pretreatment mitigated these abnormalities, suggesting protection of cardiac electrophysiological integrity through antioxidative means, consistent with the work of Priyadarsini et al. [66].

Serum biomarkers CKMB and LDH, crucial indicators of myocardial injury^76^, were elevated following adrenaline administration, affirming findings by Muders, Neubauer^77^ and Kannan and Quine^78^. Ea administration significantly reduced these markers, reflecting improved cardiomyocyte membrane stability and reaffirming its cardioprotective role.

The lipid profile (cholesterol and triglycerides) remained unaffected in this study, which contradicts Maduka, Neboh^79^, likely due to differences in treatment duration. Similarly, Ea did not significantly alter lipid levels in this context, unlike findings by Shiojima, Takahashi^80^ and Panchal, Ward^81^, again highlighting the influence of dosage and treatment length on study outcomes.

Significant renal dysfunction, marked by increased serum urea, uric acid, and creatinine levels following adrenaline exposure, aligns with findings from Salama, Mansour^82^ and Quan, Walser^83^. On the contrary, Bellomo, Wan^84^ reported no significant renal changes, highlighting contradictory clinical evidence. Our results confirm renal protective effects of Ea, as supported by Chao, Mong^85^, suggesting an ability to reverse adrenaline-induced nephrotoxicity.

Ea also demonstrated hepatoprotective potential by reducing ALT and AST levels elevated by adrenaline, as reported in agreement with Abdullah, Jarjees^86^ and Mehrzadi, Fatemi^87^. These results emphasize Ea’s capacity to prevent hepatic oxidative damage, likely contributing to its systemic protective effects.

Markers of oxidative stress showed that adrenaline elevated MDA and TOS while reducing TAC and GSH, supporting the findings of El-Marasy, El Awdan^74^ and Yüce, Ateşşahin^88^. These oxidative changes were substantially reversed by Ea, highlighting its antioxidative efficacy.

Mechanistically, oxidative stress is known to activate NRF2, stimulating pro-inflammatory cytokines and adhesion molecules^89–91^. Adrenaline increased levels of IL-1β, IL-6, and TNF-α, all of which were significantly reduced in Ea-pretreated groups, suggesting that Ea downregulates inflammatory cascades through NRF2 modulation. Elevated NRF2 expression in Ea groups underscores its anti-inflammatory impact, confirmed via histological and immunohistochemical examination. NRF2 activation has been widely acknowledged as a cellular defense response to redox imbalance^92,93^. As part of the Keap1-Nrf2 system, NRF2 is sequestered by Keap1 under normal conditions and released under oxidative stress via cysteine oxidation^94–100^. Our molecular docking analysis highlighted that Ea possesses high affinity towards the Keap1-binding pocket with a docking score comparable to known NRF2 activator CHEMBL5199314. Notably, Ea interacts with critical residues of the Keap1-NRF2 interface, suggesting a potential role in disrupting this interaction and promoting nuclear translocation of NRF2, a key step in activating antioxidant gene expression^101^. Furthermore, the PI3K/AKT signaling pathway, often associated with inflammation and cellular growth^102–104^, was significantly upregulated by adrenaline. Ea administration downregulated both PI3K and AKT expressions in cardiac tissue alongside activation of the Keap1-NRF2 antioxidant axis, confirming its involvement in suppressing inflammatory responses, as previously shown by Huang, Lin^105^. While this observation suggests a dual anti-inflammatory and antioxidant role of EA, the relationship between these two pathways remains complex. Although literature supports potential cross-talk between PI3K/AKT and NRF2 signaling, including AKT-mediated phosphorylation and stabilization of NRF2 under certain conditions, these pathway interactions can be both context and stimulus-dependent. Our results demonstrate a correlative downregulation of PI3K/AKT and upregulation of NRF2 with EA treatment; however, a direct causal relationship based on the current data cannot be concluded. Future investigations incorporating specific pathway inhibitors or gene-silencing approaches are required to delineate whether NRF2 activation is a downstream consequence of PI3K/AKT modulation by EA or whether both pathways are independently regulated.

One limitation of the present study is the use of an acute adrenaline exposure model, limited to a 48-hour duration, which may not fully capture the complex and progressive nature of cardiotoxicity associated with chronic adrenergic stimulation. Although this model allowed for the controlled investigation of early cardiac responses to sympathetic overactivation, it does not replicate the cumulative myocardial stress and pathological remodeling observed in longer-term clinical conditions such as chronic stress, heart failure, or pheochromocytoma. Consequently, the translational relevance of the findings to chronic cardiac disease states may be constrained.

Additionally, another notable limitation PI3K/AKT signaling was assessed only at the transcript level via RT-PCR, and NRF2 localization was evaluated by immunohistochemistry; no Western blot analysis was performed to quantify protein expression. As mRNA levels do not always correlate with protein abundance or activity, this limits our ability to fully substantiate pathway modulation at the functional level. These issues have been acknowledged as limitations in the current study. Future investigations will incorporate sub-chronic or chronic adrenergic stimulation models as well as protein-level validation (e.g., Western blotting) to better reflect disease-relevant mechanisms and strengthen the molecular conclusions drawn.

Despite the promising insights gained from our molecular docking analysis, one key limitation of this study is that these computational findings are predictive and have not yet been experimentally validated. Although in silico methods can provide valuable information regarding potential mechanisms of action, confirmation through experimental approaches—such as assessing Nrf2 nuclear translocation via immunofluorescence or Western blotting—is essential for drawing definitive conclusions about pathway activation. Future studies will therefore aim to substantiate these results by evaluating established downstream target markers such as HO-1 and NQO1 using protein-based assays.

Finally A notable limitation of the present study is the absence of a positive control group treated with a well-established cardioprotective agent, such as carvedilol, N-acetylcysteine (NAC), or vitamin C. The inclusion of such a comparator would have helped to validate the experimental model and provide context for interpreting the efficacy of Ea by benchmarking its effects against standard reference treatments. Consequently, without this standard comparison, it remains challenging to determine whether Ea’s observed protective effects are robust relative to established therapies. This limitation should be addressed in future studies through incorporation of appropriate positive controls.

## Conclusions

Overall, this study clearly elucidates the multifaceted protective roles of Ea in countering adrenaline-induced toxicity through a combination of antioxidative, anti-inflammatory, and molecular pathway-modulating effects involving NRF2 and PI3K/AKT signaling axes.

## Data Availability

The authors confirm that the data supporting the findings of this study are available within the article and its histological scores.
